# Additional Thoughts on Pyorrhœa Alveolaris

**Published:** 1886-01

**Authors:** A. O. Rawls


### Additional Thoughts on Pyorrhœa Alveolaris.

But is the disease of local origin unaided by susceptability of the part through systemic conditions, or is it the reverse, viz.: Systemic peculiarities of a pathologic nature, aggravated by the presence of local and foreign irritants. This is the main question to be considered in connection with the subject if we would arrive at a solution of the question of treatment.

Now, there are many practitioners who fail to see any difference between these cases, and other somewhat similar ones, the lesions of which are due entirely to local causes, and pardon me, for I do not wish to believe it, I am afraid a few of the latter do not wish to see the difference. They are wedded to their hobby, right or wrong, and for the sake of their position on the subject, would deny and defy the most palpable and potent facts that ever obtained to make a law.

It is pertinent to inquire, now, wherein the signs and symptoms exist that would indicate any difference between what may, with at least a semblance of truth, be termed typical and non-typical cases. In the article just read, I nominate typical cases as those which have for their predisposing cause, the effects upon certain organizations of mercurial medicines or chloride of sodium. By this, it is not my intention to convey the idea, that all persons who use or have used these articles will be subject to such changes of their organism as to predispose them to the conditions which I call typical, I do mean to say, however, that all cases presenting signs and symptoms indicative of what I consider typical ones have as their predisposing cause the effects acquired or inherited of one or the other of these articles, or possibly the same element or elements of other articles of food or medicine.

The non-typical cases are those which have for the causation of certain similar signs and symptoms, local irritants upon non-predisposed tissue upon healthy tissue, upon resistive tissue and all the latter implies.

The different results following the treatment of these distinct, though somewhat similar affections may be summed up in this: That in the one, you have but to remove local external foreign irritants and the integrity of tissue, tone of function and stability of pabulum assists your efforts to a cure.

In the other, not only do you contend with salivary calculous, sanguinary calculous, catarrhal infection or peculiar fungi, but with internal local irritants, internal local weakness, and a constant supply of effete matter to tissues unable to eliminate it and return to healthful tone and action.

In the one, you remove external causation, and the density of tissue preserves itself to the point when close anastomoses of nutrient channels together with well balanced affinities of exosmotic and endosmatic interchange maintain the function of the part. In the other, the internal local predisposition exists, beckoning the external to come in and share with it the demolition of parts involved.

I fancy I hear some one say that fractured bones unite, and that soft tissues again adapt themselves to and become a part of the living whole, but is this an argument of value, does it signify anything of import in behalf of their opinion, that reproduction of nutrient currents can be established between a tooth denuded of its periosteal covering, and the gum that overlaps its lifeless surface? Is there not a very great dissimilarity in the two cases?

Dr. A. W. Harlan, of Chicago, being called for, said there was some misapprehension as to his reading a paper, and that he did not know until a week before the meeting that he had been placed upon the programme to read a paper, and that after having heard Dr. Rawls, he felt like taking his "grip-sack" and going home.

Dr. Harlan then read the following short paper, which, he said, was more in the nature of notes:

